# Application of radiomics in diagnosis and treatment of lung cancer

**DOI:** 10.3389/fphar.2023.1295511

**Published:** 2023-11-01

**Authors:** Feng Pan, Li Feng, Baocai Liu, Yue Hu, Qian Wang

**Affiliations:** ^1^ Department of Radiation Oncology, China-Japan Union Hospital of Jilin University, Changchun, China; ^2^ Department of CT, Jilin Province FAW General Hospital, Changchun, China; ^3^ Department of Biobank, China-Japan Union Hospital of Jilin University, Changchun, China

**Keywords:** lung cancer, radiomics, diagnosis, artificial intelligence, NSCLC

## Abstract

Radiomics has become a research field that involves the process of converting standard nursing images into quantitative image data, which can be combined with other data sources and subsequently analyzed using traditional biostatistics or artificial intelligence (Al) methods. Due to the capture of biological and pathophysiological information by radiomics features, these quantitative radiomics features have been proven to provide fast and accurate non-invasive biomarkers for lung cancer risk prediction, diagnosis, prognosis, treatment response monitoring, and tumor biology. In this review, radiomics has been emphasized and discussed in lung cancer research, including advantages, challenges, and drawbacks.

## Introduction

Lung cancer is the most widespread cancer worldwide and is the primary cause of cancer-related deaths. As per the latest global cancer statistics report, in 2020, there will be about 2.2 million new cases of lung cancer (making up around 11.4% of all cancers) and approximately 1.8 million deaths (constituting about 18.0% of all cancers) (Pishgar et al., 2018; Sung et al., 2021). Hence, timely diagnosis and treatment continue to be crucial for enhancing the survival rate of individuals with lung cancer.

The advancement of imaging technology has led to a rapid increase in medical imaging data for diagnosing, staging, planning treatments, and evaluating responses in lung cancer patients. While conventional interpretations provide insights into lung cancer characteristics, researchers have demonstrated that a considerable amount of biological and prognostic information remains concealed within the images. Radiomics pertains to the extraction of numerous high-dimensional quantitative image features from images, creating a database for analysis (Lambin et al., 2012). Through radiomics, exceptionally valuable cancer-related information can be captured, which might be overlooked or not discernible to the naked eye.

**FIGURE 1:**
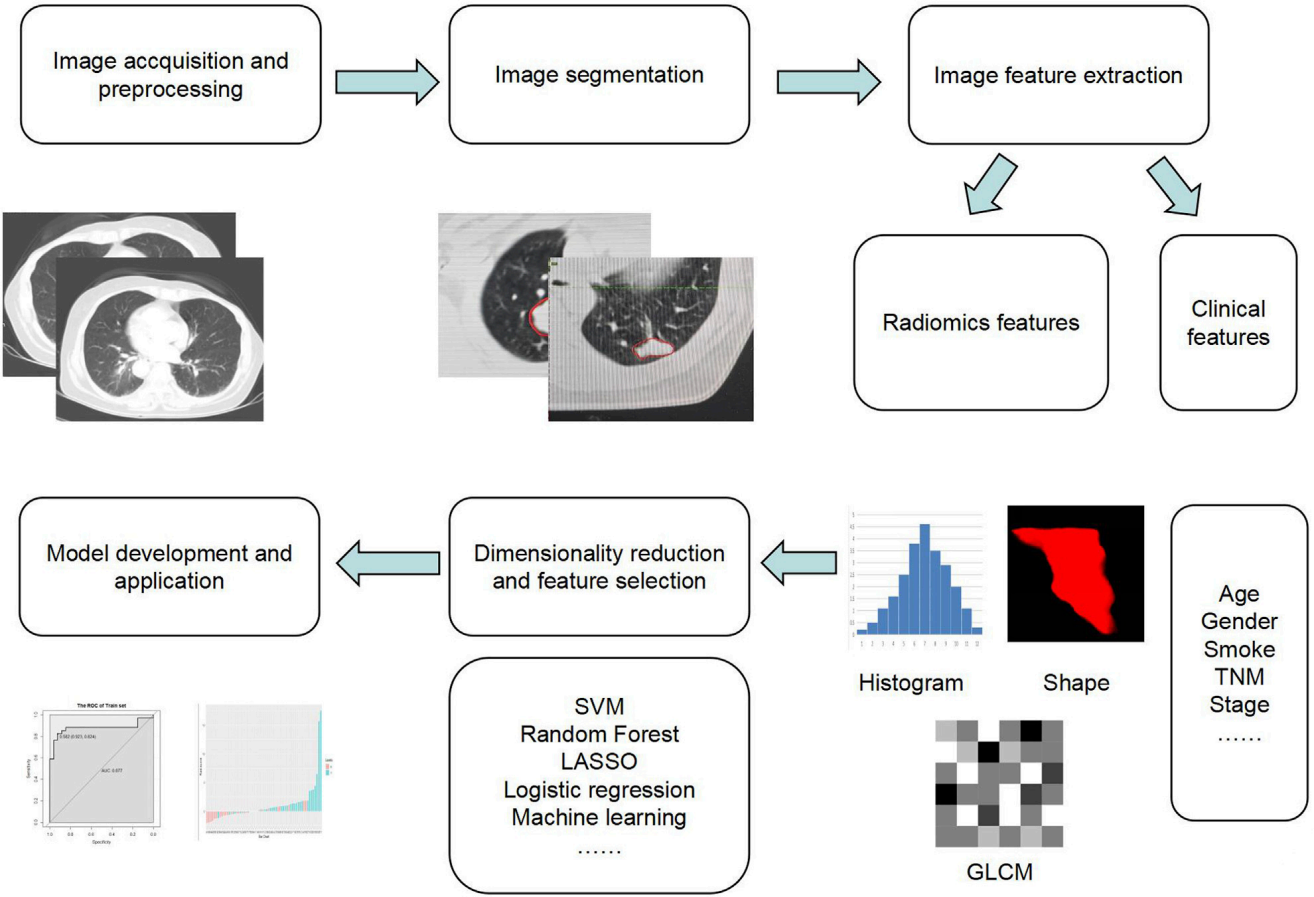
Visualization of the steps in the radiomics workflow.

The notion of radiomics was initially introduced by Dutch scholar Lambin (Lambin et al., 2012) in 2012. It involves the extraction of an array of features from medical images and employs statistical and machine learning techniques to identify the most significant imaging features for clinical information analysis, disease identification, tumor grading, and staging. Imaging technology has overcome the limitation of solely relying on subjective image interpretations by physicians, significantly enhancing the practical value of medical images in clinical settings. The process generally encompasses five steps (Mayerhoefer et al., 2020; McCague et al., 2023): image acquisition and preprocessing, image segmentation, image feature extraction, dimensionality reduction and feature selection, model development, and application. The advantage of this non-invasive examination lies in radiomics features reflecting not only the tissue’s visible characteristics but also its cellular and molecular attributes, thus offering a more comprehensive understanding of the entire tumor. Imaging features can also serve as an objective and quantitative biomarker for distinguishing between different types of tumors, analyzing tumor characteristics, and predicting prognosis. The radiomics workflow was summarized in Figure 1.

## Diagnosis

Distinguishing between benign and malignant pulmonary nodules solely based on clinician experience is challenging. Radiomics-driven qualitative diagnosis of pulmonary nodules assists clinicians in determining optimal treatment plans. Scholars have recently explored using multiple classifiers to combine model pairs for predicting primary pulmonary solid nodule malignancy. Kamiya et al. (Kamiya et al., 2014) initiated this exploration, encompassing 93 pulmonary nodules (72 malignant and 21 benign). They computed kurtosis and skewness from density histograms, revealing higher kurtosis in malignant than benign nodules. ROC curves displayed substantial area under the curve values (0.71–0.83). Choi W et al. (Choi et al., 2018) established an SVM-LASSO model using radiomics for low-dose CT-based pulmonary nodule prediction. Lesions were identified in the American College of Radiology’s Lung CT screening reporting and data system (Lung-RADS). The SVM-LASSO model achieved higher accuracy (84.1%) than the Lung-RADS model (72.2%). Chen et al. (Chen et al., 2018) extracted radiomics features for differentiating 42 malignant and 33 benign pulmonary nodules. Employing leave-one-out cross-validation, they selected four highly correlated features through sequential forward selection (SFS), constructing a support vector machine (SVM) prediction model. This SVM model achieved 84.0% accuracy, 92.9% sensitivity, and 72.7% specificity. Tu et al. (Tu et al., 2018) discovered that greater average standard deviation in CT scans corresponded to reduced uniformity and elevated “entropy,” indicating a higher likelihood of benign nodules. Their Logistic regression classifier reached peak accuracy at 79%. This classifier outperformed the LUNGRADS system established for lung cancer screening. Follow-up examinations diagnosed more ground glass nodules, especially mixed ones, as malignant rather than solid nodules. Balagurunathan et al. (Balagurunathan et al., 2019) analyzed low-dose CT images from the US National Lung Screening Trial database and determined that radiomics features improved nodule detection accuracy compared to size and shape descriptions in conventional images. The highest AUROC reached 0.83 by combining features from all categories. Beig et al. (Beig et al., 2019) conducted a radiomics study on 290 patients with lung adenocarcinoma and granuloma presenting as solitary lung nodules. They evaluated the capability of imaging features to distinguish adenocarcinoma and granuloma using features from the nodule’s surroundings, internal features, and combined surrounding and internal features. The results displayed AUC values of 0.74 for surrounding nodule features, 0.75 for internal nodule features, and 0.80 for combined surrounding and internal features. Huang et al. (Huang et al., 2018) performed a matched case-control study using NLST data to evaluate the value of a novel computer-aided diagnosis (CAD)algorithm that analyzes texture features of nodules as well as surrounding lung tissues. In the validation cohort, the area under the receiver operating characteristic curve for CAD was 0.9154. The sensitivity, specificity, PPV, and negative predictive value of CAD and the three radiologists’ combined reading were 0.95, 0.88, 0.86, and 0.96 and 0.70, 0.69, 0.64, and 0.75, respectively. Gao et al. (Gao et al., 2020)extracted 1,344 3D texture features from pulmonary nodules, achieving a classifier sensitivity of 98% for distinguishing benign and malignant nodules using support vector machines. This sensitivity was markedly higher than that of three clinicians (73% specificity). Sun et al. (Sun et al., 2017) compared the efficacy of CNN, DBN, autocoding, transfer learning, and radiomics methods in distinguishing benign and malignant pulmonary nodules. CNN exhibited superior performance, achieving the highest AUC of 0.899 ± 0.018. Zhang et al. (Zhang et al., 2020a) combined SE network and ResNeXt to form SE-ResNext, significantly enhancing feature discriminative power. This fusion achieved an accuracy of 91.67% for distinguishing benign and malignant pulmonary nodules, with an AUC as high as 0.960. Another study by Cherezov et al. (Cherezov et al., 2018) extracted radiomic features from baseline and follow-up screens and performed size-specific analyses to predict lung cancer incidence using three nodule size classes. Once used images and data from the National Lung Screening Trial (NLST), malignancy prediction accuracy was improved from 74.7% to 81.0% by implementing nodule size–specific models. Zhao et al. (Zhao et al., 2021a) classified pulmonary cryptococcosis and lung adenocarcinoma in patients with solitary pulmonary solid nodules, constructing a nomogram model incorporating clinical features, imaging signs, and radiomics scores. The AUC values were 0.91 and 0.89, respectively, facilitating clinical decision-making. Radiomics can effectively capture distinctions between preinvasive and invasive lesions within pure ground glass nodules (pGGN). Building a classification model based on radiomics features enhances the preoperative prediction accuracy of pGGN’s pathological aggressiveness. For instance, SVM, naive Bayes classifier, and logistic regression classifier demonstrated AUCs of 0.822, 0.848, and 0.874, respectively. Conversely, AUCs derived from lesion size and average CT value predictions stood at mere 0.726 and 0.786 (Wang et al., 2017). Shen et al. (Shen et al., 2020) employed SVM, random forest (RF), logistic regression (LR), extreme learning machine, and K-nearest neighbor (KNN) algorithm, post feature selection. They constructed five classifier models, then fused all prediction outcomes to determine nodule nature. This approach, incorporating texture, wavelet, and gray level features, significantly facilitated distinguishing between benign and malignant solid pulmonary nodules, achieving an accuracy of 92% (summarized in Table 1).

**TABLE 1:** Radiomics studies in early detection and lung cancer screening.

Study	Image modality	Dataset	Analytical method	Single center or multi- center	Reported performance
Kamiya et al. (2014)	CT	93 patients (72 malignant and 21 benign)	Unknown	Single	AUC 0.71-0.83
Choi et al. (2018)	CT	72 patients (31 benign and 41 malignant)	SVM + LASSO	Single	AUC of 0.84
Chen et al. (2018)	CT	76patients (42 malignant and 33 benign)	SVM	Single	AUC of 0.84
Tu et al. (2018)	CT	122patients (74 malignant and 48 benign)	logistic classification	Single	AUC of 0.79
Balagurunathan et al. (2019)	CT	588 patients (196 maliganant and 392 benign)	linear classifiers	Single	AUC of 0.83
Beig et al. (2019)	CT	290 patients (145 maliganant and 145 benign)	CNN	Single	AUC of 0.8
Huang et al. (2018)	CT	186 patients (90 maliganant and 96 benign)	Random forest classifier	Single	AUC of 0.91
Gao et al. (2020)	CT	285patients (223 maliganant and 62 benign)	SVM	Single	AUC of 0.73
Sun et al. (2017)	CT	1,018 cases from Lung Image Database Consortium (LIDC) public lung cancer	CNN	multi- center	AUC of 0.89
Zhang et al. (2020a)	CT	1,004 cases (450 malignant and 554 benign) nodules from Lung Nodule Analysis	Deep learning	multi- center	AUC of 0.96
Cherezov et al. (2018)	CT	160 incidence cases and 307 nodule‐positive controls	Deep learning	Single	AUC of 0.94
Zhao et al. (2021a)	CT	426patients (213 maliganant and 213 benign)	LASSO	Single	AUC of 0.91
Wang et al. (2017)	CT	102patients (42 maliganant and 60 benign)	logistic classification 、SVM	Single	AUC of 0.72
Shen et al. (2020)	CT	342patients (171 maliganant and 171 benign)	five classifier models	Single	AUC of 0.92

### Tumour histology

According to the 2015 WHO histological classification for lung cancer (Wu et al., 2016), lung cancer can be categorized into 9 types. In instances where CT-guided needle biopsy is not feasible, postoperative pathological sections are the sole means of obtaining pathology data. Presently, there’s no non-invasive method for pathologic classification before biopsy or surgery. In contrast to pathology, CT offers affordability and non-invasiveness, with radiomic analysis through artificial intelligence’s extensive data providing a preoperative opportunity for classifying pathology types. Oncologists consistently strive to discern cancer cell subtypes in lung cancer patients. Wu et al. analyzed 440 radiological features from CT images of NSCLC to classify ADC vs SCC, achieving an accuracy of 0.7 and an AUC of 0.72 (Haga et al., 2018). Haga et al. utilized a volume of interest method to categorize early-stage NSCLC subtypes among different observers, with averaged AUC values at 0.725 ± 0.070 (Zhu et al., 2018). Zhu et al. employed the LASSO logistic regression model, selecting five key features to create a radiomic signature for histological subtype classification. In validation cohorts, the AUC for differentiating lung adenocarcinoma (ADC) vs squamous cell carcinoma (SCC) was 0.893 (Lu Lin et al., 2019). A multiphasic CECT study displayed AUC values of 0.857, 0.855, and 0.864 for radiomics models in classifying ADC vs SCC in non-enhanced, arterial, and venous phases, respectively (Wu et al., 2016). Wu et al. (Lu Lin et al., 2019) extracted 5 image omics features to build a predictive model, yielding an AUC of 0.72. Linning et al. extracted 3 radiomic features, resulting in AUC values of 0.801, 0.834, and 0.864. Lu et al. reported radiomics models’ diagnostic performance (AUC) as 0.741 (SCLC vs NSCLC), 0.822 (AD vs SCLC), 0.665 (SCC vs SCLC), and 0.655 (AD vs SCC) in histopathologic lung cancer subtype classification (Wu et al., 2016). Chen et al. (Chen et al., 2020a) explored CT images of peripheral lung cancer using histogram-based features (max, min, mean, range, entropy, variance, skewness, kurtosis), gray co-incidence matrix, gray stroke length matrix, gray size area matrix, and neighborhood gray tone difference matrix. The CT radiology model based on neural network classification effectively distinguished SCLC (n = 35) from NSCLC (n = 34), yielding an AUC of 0.93.

### Tumour staging

Previous studies have explored the role of AI in lung cancer staging, encompassing the prediction of lymph node and distant metastasis based on primary tumor characteristics. In a study involving 657 NSCLC patients (He et al., 2017), researchers established a preoperative CT radiomics signature with a commendable capability to forecast postoperative pathological staging. The radiomics prediction model’s AUCs in the training and validation sets were 0.715 and 0.724, respectively, showcasing its value in identifying early and advanced NSCLC patients preoperatively. Aerts et al. (Aerts et al., 2014) extracted 440 image features from CT data of 1,019 lung and head and neck cancer patients, noting a correlation of 0.6 between texture features and TNM staging in lung cancer, potentially aiding in auxiliary detection for tumor staging. Chen’s work integrated gross and 6 mm peritumoral volume (GPTV6) radiomics features and independent clinical predictors into a nomogram, effectively predicting lymph node metastasis and prognosis in clinical stage IA NSCLC patients, achieving an AUC of 0.85 (Chen et al., 2023). Zhao proposed a cross-modal deep learning system successfully blending clinical knowledge and CT images into a 3D neural network, achieving an AUC of 0.926 for predicting LN metastasis in clinical stage T1 lung cancer (Pommier et al., 2010). Sushant’s approach integrated clinical parameters and CT radiomics from GTV, PTV, and LN, enhancing the predictive power of the nomogram in cT1N0M0 adenocarcinoma patients, with an AUC of 0.79 (95% CI 0.66–0.93) in external validation (Das et al., 2021). Yoshihisa investigated CT-based radiomics with AI for predicting pathological lymph node metastasis in clinical stage 0–IA NSCLC. Multivariate analysis revealed that clinical stage IA3, solid part size, and average solid CT value were independently associated with pN. The ROC yielded 0.761, with a sensitivity, specificity, and negative predictive values of 69%, 65%, and 94% in the entire cohort, respectively (Shimada et al., 2023). Yuan’s findings (Yuan et al., 2022) revealed that both the enhanced and unenhanced CT radiomics models had five features with the potential to predict hilar and mediastinal lymph node metastases in solid nodular lung cancer, achieving AUCs of 0.811 and 0.803, respectively. Cong utilized a venous computed tomography radiomics model for lymph node metastasis prediction in non-small cell lung cancer, yielding a validation group AUC of 0.73 (95% CI: 0.70–0.76) (Cong et al., 2020). Zhang (Zhang et al., 2023) demonstrated that a nomogram integrating multi-view radiomics, deep learning, and clinical features efficiently quantitatively predicted presurgical N2 diseases in clinical stage I-II NSCLC patients. Combined models displayed superior diagnostic performance compared to models using only clinical or image risk factors (AUC for combined models was 0.88). Wang’s work indicated that radiomic signatures from gross tumor volume (GTV) and peritumoral volume (PTV) exhibited strong predictive capabilities for LN metastasis, achieving AUCs of 0.829 (95% CI, 0.745–0.913) and 0.825 (95% CI, 0.733–0.918). The radiomic nomogram attained an AUC of 0.869 (95% CI, 0.800–0.938), facilitating convenient preoperative LN metastasis prediction in T1 peripheral lung adenocarcinomas (Wang et al., 2019a). Ma’s research introduced a deep learning signature based on Swin Transformer, achieving AUCs of 0.948–0.961 in predicting LN metastasis. Calibration curves indicated good fit between the DL signature’s predicted probabilities and observed LN metastasis probabilities (Ma et al., 2023). Zheng identified five CT radiomic characteristics significantly correlated with LNM. The radiomic nomogram, incorporating these characteristics along with RDW and CT-based LN status, displayed satisfactory discrimination and calibration in training (AUC 0.79; 95% CI 0.69–0.89) and validation cohorts (AUC 0.70; 95% CI 0.50–0.89) (Zheng et al., 2022). Yang et al. (Yang et al., 2018)combined preoperative venous phase enhanced CT images’ radiomics features with clinical features, achieving AUC values of 0.911 and 0.871 for predicting lymph node metastasis. Ferreira Junior et al. (Ferreira-Junior et al., 2020) calculated various radiomic features significantly associated with distant metastasis, nodal metastasis, and histology, yielding AUC values of 0.92 and 0.84 for predicting M and N stages, respectively. Fabian’s research showcased that PET/CT image radiomics features and transfer-learning deep radiomics features could predict non-invasive N-staging with the best outcome [AUC 0.871 (0.865–0.878)] using the random forest model (Laqua et al., 2023). The PET/CT-based radiomics nomogram exhibited predictive ability for occult lymph node metastasis in NSCLC, with AUCs of 0.884 in the training set and 0.881 in the testing set (Qiao et al., 2022).

On the other hand, certain studies have aimed to directly analyze lymph nodes for distinguishing between benign and malignant conditions. For instance, Bayanati et al. (Bayanati et al., 2015) conducted radiomics analysis of mediastinal lymph nodes in lung cancer patients, discovering that the fusion of texture and morphological features could enhance N staging accuracy (AUC = 0.87). Moitra et al. (Moitra and Mandal, 2019) explored convolutional neural networks (CNN) and combined CNN with recurrent neural networks (RNN) for automatic AJCC staging of non-small cell lung cancer, achieving an accuracy of 92.91%. Xie’s work introduced a PET/CT nomogram that integrates Rad-Score and SUVmax, improving LN metastasis diagnosis in non-small cell lung cancer (NSCLC) patients. The training cohort displayed an AUC of 0.881 (95% CI, 0.834–0.928), while the testing cohort demonstrated an AUC of 0.872 (95% CI, 0.797–0.946) (Xie et al., 2021).

### Tumour genotype

Lung cancer patients harboring gene mutations can significantly benefit from targeted therapy. However, the current approach to detecting gene mutation status relies primarily on biopsy or cytological analysis, which is invasive. Moreover, challenges such as biopsy sampling errors, improper procedures, patient non-compliance, and sampling difficulties can impede obtaining sufficient samples for testing. Given these limitations, there’s a need for a reproducible, straightforward, safe, and non-invasive method to preoperatively determine gene mutation status. Radiomics enables the extraction of tumor features from medical images in a high-throughput manner, revealing aspects not discernible by the human visual system. In clinical applications, radiomics models hold promise in predicting the mutation status of lung cancer genes, furnishing image-derived biomarkers for personalized targeted therapy.

### Gene mutation

Currently, imaging omics studies for predicting lung cancer gene mutations primarily focus on the EGFR gene mutations, which are the most prevalent mutations in clinical practice. The mutation rates stand at 40%–50% for Asian NSCLC patients and 10%–20% for non-Asian patients (Lilenbaum and Horn, 2016; Liu et al., 2016). Aerts et al. (Aerts et al., 2016) non-invasively extracted data from pre-treatment CT images of lung cancer patients to assess EGFR mutation status and predict tumor response to targeted drugs like gefitinib. Their findings indicated that tumor volume, texture, and gradient characteristics could predict EGFR mutation status. Tu et al. examined 404 NSCLC patients and found that radiomics signatures exhibited superior performance (AUC = 0.762 and 0.775 in training and validation cohorts) compared to clinical and morphological features in predicting EGFR mutations (Tu et al., 2019). Similarly, Lu et al.'s retrospective study involving 104 patients demonstrated that radiomic models (AUC = 0.837) outperformed qualitative CT feature-based models (AUC = 0.768) (Lu et al., 2020). The integration of clinical and morphological image features further enhanced model performance with increased AUC values (Tu et al., 2019; Lu et al., 2020). Rios et al. (Rios Velazquez et al., 2017) reached a similar conclusion in their study, discovering correlations between EGFR mutation status and certain features in wavelet analysis and gray co-occurrence matrix. They explored the potential connection between high-dimensional microscopic features and tumor image morphological characteristics. Texture features like light texture furrows and uniform overall gray distribution were correlated with EGFR mutation. Numerous studies have investigated these associations. Wang et al. (Wang H. et al., 2019) developed a predictive model to distinguish between EGFR with exon 21 mutation and wild-type lung cancer, achieving accuracy, sensitivity, and specificity rates of 0.87, 0.946, and 0.738, respectively. Zhang et al. (Zhang et al., 2020b) investigated PET-CT images of 248 NSCLC patients before treatment, observing a relationship between lower peak normalized intake value and EGFR mutation. By extracting texture features from PET-CT images and combining metabolism and structure texture features, they established an EGFR mutation prediction model using logistic regression. The AUC in the training set was 0.79, and in the validation set, it reached 0.85, confirming PET-CT’s value in predicting EGFR mutation types in NSCLC patients. Yipp et al. (Yip et al., 2017) discovered PET imaging features capable of detecting EGFR mutation status, but no features linked to KRAS mutation were identified.

Liu et al. discovered that 3D radiomics features from 11 lung adenocarcinoma tissues correlated with EGFR mutations, and the integration of relevant CT feature parameters and clinical features significantly enhanced EGFR prediction accuracy (Liu et al., 2016). Rios et al. demonstrated that CT texture features in lung adenocarcinoma patients could effectively differentiate between EGFR and KRAS mutated gene positivity (AUC = 0.80) (Rios Velazquez et al., 2017). Agazzi et al. (Agazzi et al., 2021) formulated a CT-based classification model to distinguish EGFR mutated, ALK mutated, and non-mutated tumors. They discovered that mutation types were associated with skewness, with EGFR mutated tumors displaying the highest skewness, while ALK rearranged tumors exhibited the lowest value. Tumors without mutations displayed median values, resulting in an accuracy of about 82% for the model. Wang et al. (Wang et al., 2019c) examined 61 pulmonary nodules in 51 patients with early lung adenocarcinoma. They selected 13 image omics features and employed a support vector machine (SVM) to predict tumor mutation load and the status of certain driving mutations (EGFR and TP53) in early lung adenocarcinoma patients. The model demonstrated feasibility and effectiveness, with the potential for further improvement when combined with clinical data. SHIRI et al. (Shiri et al., 2020) identified the potential of imaging features in predicting and identifying EGFR and KARS gene mutations in 150 NSCLC patients. Some researchers suggest that traditional CT imaging signs could aid in predicting EGFR mutation presence in advanced lung adenocarcinoma.

Yamamoto et al. (Aerts et al., 2016) identified correlations between signs like tumor location and significant pleural effusion with ALK gene mutation. Rizzo et al. (Rizzo et al., 2016) found that pleural effusion presence was associated with ALK gene mutation, while tumor location remained independent of ALK mutation. Song et al. demonstrated that radiomics-derived machine learning models could identify ALK mutations in lung adenocarcinoma with 76% accuracy (Song et al., 2020). Yoon et al. (Yoon et al., 2015) exhibited the ability to identify adenocarcinomas with ALK, ROS1, and/or RET fusion phenotypes using CT and PET imaging, achieving a sensitivity of 0.73 and specificity of 0.70. Prediction was achievable using CT radiomics, achieving an AUROC of 0.914 when combined with clinical and CT semantic features, surpassing performance based solely on clinical (0.735) or radiomic features (0.890) (Hao et al., 2022). The PET-CT radiomics model achieved an AUROC of 0.88, with no improvement observed upon incorporation of clinical features (Hao et al., 2022) (summarized in Table 2).

**TABLE 2:** Radiomics studies in NSCLC with an aspect of biology.

Study	Image modality	Targeted gene	Dataset	Analytical method	Reported performance
Aerts et al. (2016)	CT	EGFR	47 patients with early-stage NSCLC	Logistic regression	AUC = 0.67
Tu et al. (2019)	CT	EGFR	404 patients with NSCLC (243 cases in the training cohort and 161 cases in the validation cohort	Mann-Whitney U test or Chi- square test	AUC = 0.79
Lu et al. (2020)	CT	EGFR	104 lung adenocarcinoma patients	Logistic regression	AUC = 0.83
Rios velazquez et al. (2017)	CT	EGFR	763 lung adenocarcinoma patients	Wilcoxon test	AUC = 0.69
Wang et al. (2019a)	CT	EGFR	309 lung adenocarcinoma patients	Mann-Whitney U test or Chi- square test	AUC = 0.78
Zhang et al. (2020b)	PET/CT	EGFR	248 NSCLC patients	LASSO	AUC = 0.85
Yip et al. (2017)	PET/CT	EGFR	348 NSCLC patients	Wilcoxon rank-sum test	AUC = 0.5
Agazzi et al. (2021)	CT	EGFR and ALK	84lung adenocarcinoma patients	ANOVA	AUC = 0.817
Wang et al. (2019b)	CT	EGFR and TP53	51 early stage lung adenocarcinoma patients	SVM	AUC = 0.606
Shiri et al. (2020)	PET/CT	EGFR and KRAS	211NSCLC patients	Machine Learning	AUC = 0.69
Song et al. (2020)	CT	ALK	335 lung adenocarcinoma patients	Mann-Whitney U test or Chi- square test	AUC = 0.80–0.88
Rizzo et al. (2016)	CT	EGFR, K- RAS, and ALK	285 NSCLC patients	Wilcoxon test	AUC = 0.82,0.87,0.65
Song et al. (2020)	CT	ALK	335 lung adenocarcinoma patients	Mann-Whitney U test or Chi- square test	AUC = 0.83–0.88
Yoon et al. (2020)	CT	ALK, ROS 1and RET	539 lung adenocarcinoma patients	*t*-test or Chi- squared test	sensitivity and specificity,0.73and0.7 0
Yoon et al (2022)	CT	ALK	193 patients with NSCLC	SVN	AUC = 0.914

### Immune microenvironment and tumor mutation burden

Radiomics analysis offers a non-invasive means to comprehensively assess the entire tumor in medical images, capturing tumor characteristics. Some studies have demonstrated that baseline imaging omics analysis can effectively predict PD-L1 expression levels and TMB status. Tian et al. (Tian et al., 2021) performed radiomics analysis on CT scans of 939 NSCLC patients with stages IIB to IV prior to ICIs treatment, constructing a model to evaluate PD-L1 expression. In the training, validation, and test sets, the model predicted high PD-L1 expression (>50%) with AUC values of 0.78, 0.71, and 0.76, respectively, aiding in identifying NSCLC populations likely to benefit from immunotherapy. He et al. (He et al., 2020) divided a dataset of 327 NSCLC patients with known TMB status into high and low TMB groups based on a critical value of 10 mutations per Mb base. They established a TMB radiomic biomarker (TMBRB) to distinguish between these groups, with AUC values of 0.85 and 0.81 in the training and test sets, respectively. Subsequently, Wen et al. (Wen et al., 2021) included 120 advanced NSCLC patients and developed a multimodal prediction model for PD-L1 expression and TMB status by combining clinical factors with CT morphological and baseline imaging features, outperforming the simple radiomics model. The AUC values for predicting PD-L1 expression in the training and test sets were 0.839 and 0.793, respectively, and for predicting TMB status, they were 0.818 and 0.786. Radiomics-based prediction of PD-L1 positivity achieved an AUROC of 0.76 for PD-L1 ≥ 50%. However, due to the lack of consensus on PD-L1 expression cut-offs, lower thresholds are often used in clinical practice, such as ≥ 1% (Hellmann et al., 2019; Mok et al., 2019). Another study developed a CT-radiomics model for different PD-L1 levels, achieving AUROC of 0.950, 0.934, and 0.946 for PD-L1 < 1%, 1–49%, and≥50%, respectively (Wang et al., 2022). Since PD-L1 expression is dynamic and variable, adopting a fixed threshold is considered overly simplistic and susceptible to data variability depending on the IHC assay used (Tsimafeyeu et al., 2020).

### Clinical outcome prediction

Predicting treatment response or prognosis is challenging in lung cancer. Radiomics has been successfully employed to predict the prognosis of lung cancer patients undergoing surgery, radiation therapy, or targeted therapy. Crucial prognostic factors include local recurrence and distant metastasis. Effectively predicting risk factors for these outcomes holds significant importance.

### Radiotherapy

The assessment of radiotherapy sensitivity and prediction of radiation-induced damage are essential for tailoring individualized treatment plans for lung cancer patients. Huynh’s study demonstrated that certain pre-treatment CT radiomics features could predict the efficacy of stereotactic radiotherapy for patients with stages I-II non-small cell lung cancer, outperforming traditional CT indicators (Huynh et al., 2016). Paul et al. developed a multiple regression model utilizing four radiomics features to predict radiotherapy treatment effects in 122 patients with stages I-II non-small cell carcinoma, aiming to minimize side effects and shorten treatment durations. Mattonen et al. found that regular CT radiomics analysis could detect subtle changes that might go unnoticed by the human eye when lung cancer recurred, enabling differentiation from imaging changes due to radiation injury (Mattonen et al., 2016). Paul et al. demonstrated a significant correlation between changes in daily CT characteristic parameters of radiotherapy patients and radiation-induced damage (Paul et al., 2017). In summary, by establishing relevant clinical models based on CT radiomics data, individualized radiotherapy guidance can be provided for lung cancer patients, enhancing the safety of their radiotherapy.

For patients who are not suitable candidates for surgery, SABR is the recommended treatment for peripherally located stage I NSCLC, and if SABR is not available, a hypofractionated radiotherapy regimen with a high biologically equivalent dose is advised. Stereotactic body radiation therapy (SBRT) is the preferred radiation treatment modality for lung cancer, particularly for small local lesions in inoperable patients (Postmus et al., 2017; Pentheroudakis and Committee, 2020). Radiomic features have demonstrated the capability to predict outcomes in SBRT-treated patients that conventional imaging measures may not foresee (Huynh et al., 2016). Numerous studies have aimed to predict various clinical endpoints such as local control, disease-free survival, and overall survival with remarkable accuracy (Hosny et al., 2018; Yu et al., 2018)^.^ For SBRT-treated NSCLC, CT deep learning features have shown potential to predict SBRT treatment failure and guide dose reduction (Lou et al., 2019). A classifier incorporating nine radiomic features, including gray level co-occurrence matrix (GLCM) texture features and first-order features, exhibited a detailed dose-response relationship at different time points after SBRT (Moran et al., 2017), particularly distinguishing between local failure and radiation-induced lung injury (RILI) (Mattonen et al., 2014). Similarly, PET-CT radiological features such as PET IC2 and CT flatness are correlated with tumor recurrence after SBRT and have demonstrated predictive capabilities for tumor recurrence in external validation, with an AUROC of 0.905 (Dissaux et al., 2020).

### Chemotherapy

The evaluation of chemotherapy efficacy for lung cancer is primarily based on the RECIST standard using CT images. However, RECIST alone may not capture early chemotherapy effects effectively (Shang et al., 2016). Pathological response after chemotherapy, based on the extent of residual tumor tissue, better reflects the efficacy of chemotherapy and can predict the survival rate of lung cancer patients (Hellmann et al., 2014). Many studies have validated the application of radiomics in predicting chemotherapy outcomes for non-small cell lung cancer (NSCLC) patients. In a study involving 85 patients with resectable locally advanced (stage II-III) NSCLC, radiomics analysis was performed on thoracic primary tumors and lymph nodes to predict pathological complete response (pCR) after neoadjuvant chemoradiotherapy. Image-residual features, describing features like sphericity of the primary tumor and homogeneity of lymph nodes, were found to significantly predict pCR. Moreover, lymph node phenotype information exhibited stronger predictive power for pathological remission compared to radiomics features from the primary tumor (Coroller et al., 2017). Coroller TP examined CT imaging features of stage II to III NSCLC patients before neoadjuvant chemoradiotherapy and correlated them with tumor pathological response after surgery. Several characteristic parameters were found to be significantly associated with total pathological residual lesions (Coroller et al., 2016). CT imaging features have demonstrated a strong correlation with the degree of pathological response to chemotherapy in lung cancer patients, which can guide individualized chemotherapy approaches. For instance, CT volume features were utilized as predictors of survival in patients with limited-stage small cell lung cancer (SCLC) after chemoradiotherapy, and the maximum three-dimensional tumor diameter was significantly associated with local recurrence, distant metastasis, and overall survival (Kamran et al., 2020). Fusion of features from peritumoral regions of different distances improved the accuracy of predicting chemotherapy response in non-small cell lung cancer patients, achieving an AUROC of 0.85 (Chang et al., 2022). In patients receiving radiotherapy, CT-based radiomics, particularly the percentage of GLSZM (gray-level size zone matrix) area, demonstrated the capability to predict disease recurrence within 2 years after treatment, with an AUROC of 0.673. When combined with clinical characteristics, the AUROC further increased to 0.738 (Huynh et al., 2017). Additionally, for the prediction of efficacy in small cell lung cancer (SCLC) after chemotherapy, a radiomics signature constructed from CT images prior to and after two cycles of chemotherapy achieved an AUROC of 0.797, outperforming prediction using known clinical features (Wei et al., 2019).

### Targeted therapy

Targeted therapy has become a crucial treatment approach for lung cancer patients with driver gene mutations. Radiomics analysis of CT images has shown promise in predicting the efficacy of targeted therapies for these patients. Aerts et al. conducted a study comparing CT image characteristics of NSCLC patients before and after gefitinib treatment. By analyzing images from 47 patients with early-stage NSCLC, they extracted various imaging and volume features. The study demonstrated that radiomics features at baseline and the incremental changes in features between two examinations were predictive of EGFR mutation status. The incremental changes between these features showed the highest predictive significance, with an area under the curve (AUC) range of 0.74–0.91. This study not only highlighted the association between imaging features and tumor phenotype but also explored changes in imaging features before and after targeted therapy in NSCLC patients with different EGFR mutation statuses (Aerts et al., 2016). Another study utilized CT imaging features to predict the efficacy of crizotinib treatment in ALK rearranged NSCLC patients. This research involved extracting 481 imaging features from CT images of 63 stage IV patients. Eventually, three features were selected to construct a predictive test model for progression-free survival (PFS). The model achieved an AUC of 0.824 for predicting PFS, indicating the potential of CT imaging as a predictor for the efficacy of targeted therapies (Li et al., 2020). In conclusion, CT imaging features hold promise as predictors for individualized targeted therapy outcomes in lung cancer patients with driver gene mutations. These findings suggest that radiomics analysis of CT images can play a crucial role in guiding targeted treatment approaches for lung cancer patients.

### Immunotherapy

Valerio et al. conducted a study involving 59 NSCLC patients treated with PD-1 inhibitors and found that imaging features alone were effective in predicting treatment response. This suggests that radiomics analysis of imaging features can provide valuable insights into the efficacy of immunotherapy (Rios Velazquez et al., 2017; Yip et al., 2017). Sun et al. established a Radiomics score (RS) that could predict local CD8^+^ T lymphocyte infiltration in NSCLC patients, enabling the distinction between immune inflammatory and immune desert tumor immune types. The model achieved an AUC of 0.76 and was further correlated with ORR and OS in patients receiving immune checkpoint inhibitors. This indicates that radiomics analysis can predict the efficacy of immunotherapy by assessing the local tumor microenvironment (Sun et al., 2018). In another study by Sun et al., which included 68 patients with metastatic solid tumors receiving combination therapy of palizumab and stereotactic radiation therapy (SBRT), low RS tumors were associated with higher local tumor control failure rates and lower reactivity to SBRT. This translated to shorter median PFS and OS in patients with low RS tumors. This suggests that RS can assist clinicians in predicting which patients will benefit from combination therapy and avoid immunotherapy-related toxicity (Korpics et al., 2020). Trebeschi et al. collected data from cases of advanced malignant melanoma and NSCLC treated with ICIs. They built a machine learning classification model using radiomics features extracted from pre-treatment enhanced CT images of lesions to distinguish treatment response. The AUC value of the model for predicting treatment response in NSCLC lesions was 0.83, and a difference in 1-year survival rate between responders and non-responders was observed (Trebeschi et al., 2019). Tunali et al. retrospectively collected data from 228 NSCLC patients to predict hyperprogressive disease (HPD) occurrence. They built an HPD prediction model based on imaging features of the tumor interior and peri-tumor areas, achieving an AUC value of 0.843. Incorporating clinical variables further increased the AUC to 0.865 (Tunali et al., 2019). Vaidya et al. performed baseline radiomics analysis on 109 NSCLC patients, delineating internal and peritumoral areas of the tumor. Their HPD prediction model achieved AUC values of 0.85 and 0.96 in the training and test sets, respectively, confirming the potential of baseline radiomics markers in identifying patients at risk of HPD (Lo Russo et al., 2020; Vaidya et al., 2020). Mu et al. included 194 patients with advanced NSCLC and extracted multi-parameter imaging features from PET, CT, and PET/CT fusion images to distinguish between durable clinical benefit (DCB) and no durable clinical benefit (NDB) based on PFS. The model achieved AUC values of 0.86, 0.83, and 0.81 in the training set, retrospective test set, and prospective test set, respectively (Mu et al., 2020)These studies collectively demonstrate the utility of radiomics analysis in predicting treatment response, distinguishing immune microenvironment characteristics, and identifying patients at risk of adverse outcomes during immunotherapy for lung cancer.

### Metastases prediction

Lung cancer brain metastases account for the vast majority of adult brain metastases and are generally considered to be one of the factors with poor prognosis. Meissner et al. developed radiomics classifers allows for a non-invasive assessment of the intracranial PD-L1 expression in patients with brain metastases (BM) secondary to NSCLC with AUC of 0.84 (Meissner et al., 2023). Ahn et al. found that radiomic features of contrast-enhanced T1-weighted images (T1WIs) of BMs predict EGFR mutation status in primary lung cancer cases with the highest AUC of 0.8909 (Ahn et al., 2020). Chu et al. developed a CT radiomics BM model of predicted for BM risk stratification in NSCLC patients and the AUC was 0.84 (95% confidence interval: 0.80–0.89) (Chu et al., 2023). Chen et al. built a model of survival duration using both clinical and radiomic feature of MR imaging of BM from NSCLC and the radiomic scores enabled the separation of each mutation-positive group into two subgroups with significantly different survival durations (Chen et al., 2021). Chen’s study demonstrated that MR imaging based radiomic analysis of BM in patients with primary lung cancer may be used to classify EGFR, ALK, and KRAS mutation status and the AUC values based on cross validation was 0.912, 0.915, and 0.985, respectively (Chen et al., 2020b). Fan’s foundings suggested that multiregional radiomics of BM for predicting EGFR mutations and response to EGFR-TKI and AUC were 0.889 and 0.808 in external validation cohort respectively (Fan et al., 2023a). The research of Fan showed preoperative MRI-based radiomics could assess T790M resistance mutation after EGFR-TKI treatment in NSCLC patients with BM with AUCs of 0.860 in the external validation sets (Fan et al., 2023b). The research of Li and Niu also showed similar results (Li et al., 2022; Niu et al., 2023). Hou’s study indicated that MRI radiomics can be used to detect the EGFR mutation of hepatic metastasis of NSCLC patients, and AUCs was 0.908 in the training set and 0.884 in the training set (Hou et al., 2023). Tang et al. used radiomic features extracted from the contrast-enhanced chest CT to built a model and evaluated metastatic NSCLC patients’ prognosis in osimertinib treatment and the C-index 0.755 (Tang et al., 2021). Rahul et al. also found CT texture analysis could be used to assess the patients of metastatic NSCLC likely to benefit from nivolumab (Ladwa et al., 2020). Zhao et al. constructed the model which combined with nine selected radiomic features, could predict intracranial progression in ALK-positive NSCLC patients with BM undergoing ensartinib treatment. The Kaplan-Meier analysis showed that the progression-free survival (PFS) difference between the high- and low-risk groups distinguished by the Rad-score was significant (*p* = 0.017) (Zhao et al., 2021b).

### Survival analysis

Survival analysis investigations encompass various metrics, including overall survival (OS), progression-free survival (PFS), local relapse-free survival (LRFS), distal metastasis-free survival (DMFS), disease-free survival (DFS), and disease-specific survival (DSS). Kang et al. (Carvalho et al., 2013) discovered that the highest standard uptake value (SUV max) and OC-CSH, which signifies tumor heterogeneity, stand as pivotal prognostic factors for PFS, while OC-CSH is a significant prognostic indicator for LRFS and DMFS. Carvalho et al.'s research indicated a meaningful connection between the relative volume of the tumor containing 80% SUV and OS. Furthermore, a larger relative volume of the tumor, paired with a higher SUV, correlated with improved prognosis. Carvalho et al. (Abbas et al., 2023) validated the predictive capability of δ-radiomic features (including volume, texture features, and intensity-volume histogram [IVH]), demonstrating their correlation with OS. Van Timmeren et al.'s investigation highlighted the significance of a radiomic model based on preprocessed CT and recalibrated cone-beam CT images (concordance index = 0.69, *p* = 4.0 × 10^−10^) (Van Timmeren et al., 2016) and a combined model featuring preprocessed features and δ radiomic features (concordance index = 0.675, *p* = 1.3 × 10^−5^) in Fave et al.'s study, both proving influential in predicting OS (Fave et al., 2015). Coraller TP et al. constructed an image omics model with 635 features, 35 of which predicted metastasis and 12 of which predicted survival. The predictive capacity of imaging features surpassed that of traditional tumor volume (Peng et al., 2022). Grove et al. (Grove et al., 2015) identified heterogeneity indicators like burr and entropy as robust prognostic determinants for OS among early lung cancer patients. Aerts et al. introduced a series of features, encompassing size, shape, texture, and wavelets, capable of predicting lung cancer patient prognosis. Additional research unveiled a link between CT radiomics markers and DFS (Huang et al., 2016). Parmar et al. (Parmar et al., 2015) discerned that tumor size, intensity, shape, texture, and Baumann sign were associated with prognosis, stage, and histological type of lung cancer patients. Cherezov et al. (Cherezov et al., 2019) harnessed texture features to uncover the tumor microenvironment, revealing that the degree of tumor heterogeneity could potentially discern malignancy and aggressiveness, distinguishing long-term and short-term survival rates among lung cancer patients. Tang et al. found radiomics clinical probability-weighted model could predict prognosis for non-small cell lung cancer (NSCLC) with the AUC of 0.949 (Tang et al., 2023). The research of Francesca Botta showed that a radiomics model was able to separate high-risk and low-risk patients for OS of NSCLC and the CTs reconstructed with Iterative Reconstructions (IR) algorithm showed the best model performance (Botta et al., 2020). Hou et al. construct a deep learning model combining radiomic of contrast-enhanced computed tomography and clinical features to predict the overall survival of patients with NSCLC and AUC values of 0.76, 0.74, and 0.73, respectively, 8, 12, and 24 months after diagnosis (Hou et al., 2022). Lan et al. developed and validated a radiomics prognostic scoring system (RPSS) for prediction of progression-free survival (PFS) in patients with stage IV non-small cell lung cancer (NSCLC) treated with platinum-based chemotherapy and showed significant prognostic performance (He et al., 2021). Meanwhile, Win et al. (Win et al., 2013) evaluated tumor heterogeneity and permeability in PET/CT images, concluding that CT-derived texture heterogeneity was solely connected to survival within the radical treatment group, while the palliative care group’s survival correlated with CT-derived texture heterogeneity, tumor stage, and permeability. In summary, a correlation exists between certain radiomics features and survival indicators. Recognized texture features mirror tumor heterogeneity, a quality linked with tumor aggressiveness and an unfavorable prognosis for lung cancer.

## Discussion

Recent research indicates that radiomics holds substantial potential for the differentiation of colorectal cancer and the assessment of its prognosis. However, there remain certain limitations prior to its widespread clinical application. Firstly, standardizing image data within radiology departments becomes challenging due to variations in scanning instruments across manufacturers and diverse image acquisition protocols adopted by different medical facilities. Secondly, radiomics studies primarily source data from single centers with limited patient enrollment. Consequently, addressing how to facilitate collaborative multi-center investigations or conducting cross-center validation of single-center data models poses a significant challenge. Thirdly, the delineation of radiomics’ ROIs is often performed manually, heavily reliant on the radiologist’s expertise. Manual outlining consumes time and effort, resulting in diminished reproducibility. Fourthly, radiomics research predominantly consists of retrospective studies, lacking prospective investigations. Lastly, limitations of case enrollment. The main selection of enrolled non-small cell lung cancer cases is solid lesions with clear boundaries. Therefore, lesions with fewer solid components or pure ground glass density, lesions with cavities, and unclear boundaries with mediastinum and pleura, or lesions with concomitant consolidation and atelectasis, and lesions covered by a large amount of pleural fluid, cannot extract effective image information. Therefore, higher precision image segmentation algorithms are needed.

In recent times, considerable endeavors have been directed towards achieving standardized radiomics imaging scans. Marta Ligero’s investigation demonstrated that post-acquisition processing of CT images and normalization techniques for radiomics enhance classification accuracy (Ligero et al., 2021). Yajun Li’s work revealed that a normalization method based on generative adversarial networks (GAN) could mitigate radiomics feature variability stemming from distinct CT imaging protocols, thereby facilitating multicenter radiomics analysis (Ligero et al., 2021). Collaborations between the National Institutes of Health (NIH) and the National Cancer Institute (NCI), alongside various national healthcare institutions, have led to the establishment of standardized clinical imaging databases for employment in imaging omics research.

To address the limitations stemming from the limited patient count in single-center radiographic studies and challenges in model verification, the trend toward multi-center radiographic studies has gained traction. Fan developed a CT radiomics feature model using data from multiple centers, exhibiting enhanced performance in distinguishing adenocarcinoma from squamous cell carcinoma subtypes in NSCLC (Song et al., 2023). Liu established machine learning models based on CT images from diverse centers, proving their utility in assessing EGFR status in non-small cell lung cancer patients, with the RF model surpassing LR, DT, and SVM models (Liu et al., 2022; Ma et al., 2023). Janna’s work with FDG-PET/CT radiomics from multiple centers showcased its capacity to evaluate early treatment response in NSCLC patients (Schoenmaekers et al., 2019). Mubarik’s research demonstrated that PET-based radiomics from various centers could predict prognosis for NSCLC patients undergoing radiotherapy or chemo-radiotherapy (van Timmeren et al., 2019). The development of an optimized 18F-FDG PET/CT radiomics model, predicting EGFR mutation status and prognosis in lung adenocarcinoma, was realized through a multicenter study (Arshad et al., 2019).

More recently, the integration of machine learning, including deep learning, has progressively found its way into radiomics research. In comparison to conventional radiomics, this approach eliminates the need for image segmentation and interim feature extraction (Chartrand et al., 2017), thereby diminishing the errors linked to manual image segmentation and conserving medical resources. However, its adoption in the current literature remains limited, which might be due to its large training dataset requirement and suboptimal model interpretability.

## Conclusion

Radiomics holds substantial promise for the clinical realm of lung cancer. It proves valuable in diagnosing and distinguishing colorectal cancer, gauging disease stage, anticipating treatment responses, and enhancing prognostic insights. Although challenges and limitations persist in the extensive application of radiomics in clinical settings, as image standardization is established and machine learning techniques are further explored, radiomics is poised for widespread adoption in the near future.
